# Downregulation and pro-apoptotic effect of hypoxia-inducible factor 2 alpha in hepatocellular carcinoma

**DOI:** 10.18632/oncotarget.8952

**Published:** 2016-04-23

**Authors:** Sheng-li Yang, Li-ping Liu, Leilei Niu, Yun-fan Sun, Xing-rong Yang, Jia Fan, Jian-wei Ren, George G. Chen, Paul B.S. Lai

**Affiliations:** ^1^ Department of Surgery, The Chinese University of Hong Kong, Prince of Wales Hospital, Shatin, NT, Hong Kong, China; ^2^ Shenzhen Research Institute, The Chinese University of Hong Kong, Shenzhen, Guangdong, China; ^3^ Department of Hepatobiliary and Pancreas Surgery, The Second Clinical Medical College of Jinan University (Shenzhen People's Hospital), Shenzhen, Guangdong, China; ^4^ Department of Liver Surgery, Zhongshan Hospital & Liver Cancer Institute, Fudan University, Shanghai, China

**Keywords:** hepatocellular carcinoma, HIF-2α, overall survival, prognosis, apoptosis

## Abstract

The role of HIF-2*α* in hepatocellular carcinoma (HCC) is unclear. The aim of the present study was to investigate the expression pattern and role of HIF-2*α* in HCC patients. Immunohistochemical staining and western blotting analyses were applied to detect the protein level of HIF-2*α* in 206 paired HCC and peritumoral tissues. Kaplan-Meier survival and Cox proportional hazard regression analyses were performed to identify risk factors for overall survival and recurrence-free survival in these patients. The function of HIF-2*α* was studied in HCC cells and *in vivo* models. We found that the protein levels of HIF-2*α* in HCC tissues were lower than in peritumoral tissues, and were negatively correlated with tumor size (*P* < 0.05). Kaplan-Meier survival and univariate analysis revealed that HCC patients with high HIF-2*α* protein levels had longer overall survival (*P* < 0.05). Over-expression of HIF-2*α* induced apoptosis in HCC cells and increased the levels of pro-apoptotic proteins, Bak, ZBP-89 and PDCD4, whereas the inhibition of HIF-2*α* expression achieved opposite results. The findings were confirmed in a mouse HCC xenograft model. In conclusion, our study revealed that HIF-2*α* was decreased and played an anti-tumorigenic role in HCC.

## INTRODUCTION

As the fifth most common cancer, hepatocellular carcinoma (HCC) is one of the commonest cause for cancer-related deaths worldwide [1, 2]. It is well-known that HCC is one of the most hypervascularised tumors with rich blood perfusion; however, because of rapid cell proliferation and aberrant blood vessels formation, it still contains hypoxic regions [3]. Intra-tumor hypoxia of HCC leads to aggressive tumor progression, insensitivity to chemotherapy and radiation, more aggressive phenotype and worse prognosis [3, 4]. Tumor cells and tissues adapt to a hypoxic microenvironment through the activation of numerous hypoxia-related molecules, among which hypoxia-inducible factors (HIFs) are the most important ones [3]. Through regulating a number of target genes that play crucial roles in many aspects of tumor biology, HIFs have significant impacts on cell proliferation, tumor angiogenesis, metastasis, and resistance to chemotherapy and radiation [5, 6].

HIFs are heterodimers which consist of hypoxia-regulated-α (HIF-α) and oxygen-insensitive β subunits [7, 8]. At present, three types of α subunits have been found, HIF-1α, HIF-2α and HIF-3α [9–11]. Among them, the expression pattern and correlation between HIF-1α and liver cancer prognosis is clear. It is generally accepted that HIF-1α is highly expressed in HCC tissues and is associated with a poor prognosis of HCC patients [12, 13]. However, there are limited publications on the role of HIF-2α in HCC, and opposing findings have been reported, as both increased and decreased expression of HIF-2α in HCC tissues have been described [14, 15], and HIF-2α expression has been associated with better or worse prognosis of HCC [14–18].

In this study, we collected the data and HCC tissues from 206 patients admitted in a single medical institution for curative hepatectomy during a 16-year period and continuously followed up for at least 5 years. Specifically, The design of this study followed the Reporting Recommendations for Tumor Marker Prognostic Studies (REMARK) statements [19] and Transparent reporting of a multivariable prediction model for individual prognosis or diagnosis (TRIPOD): the TRIPOD Statement [20]. We determined the level of HIF-2α protein in HCC tumor tissues and the paired peritumoral tissues by using two kinds of HIF-2α primary antibodies, and analyzed the prognostic significance of HIF-2α for overall survival (OS) and recurrence-free survival (RFS). We hypothesized that HIF-2α was downregulated in HCC tissues and involved in the development of HCC. After validation of expression patterns and function of HIF-2α in HCC, we investigated the effects of HIF-2α on HCC cell proliferation and related mechanisms.

## RESULTS

### Patient characteristics

Among 206 patients, 4 patients had no preoperative serum α-fetoprotein (AFP) recorded in the data, and 5 patients had no preoperative serum alanine aminotransferase, bilirubin, as well as albumin level information recorded in the data. The main demographic, biochemical and clinical characteristics of the included patients are presented in Supplementary Table S1. The mean (±SD) and median (range) time for follow-up was 124.9 (±38.7) months and 117.5 (63–183) months, respectively. 126 (61.2%) patients died and 133 (64.5%) patients had recurrence until the last follow-up.

### The level of HIF-2α in HCC tissues and its clinical significance

**Figure 1:**
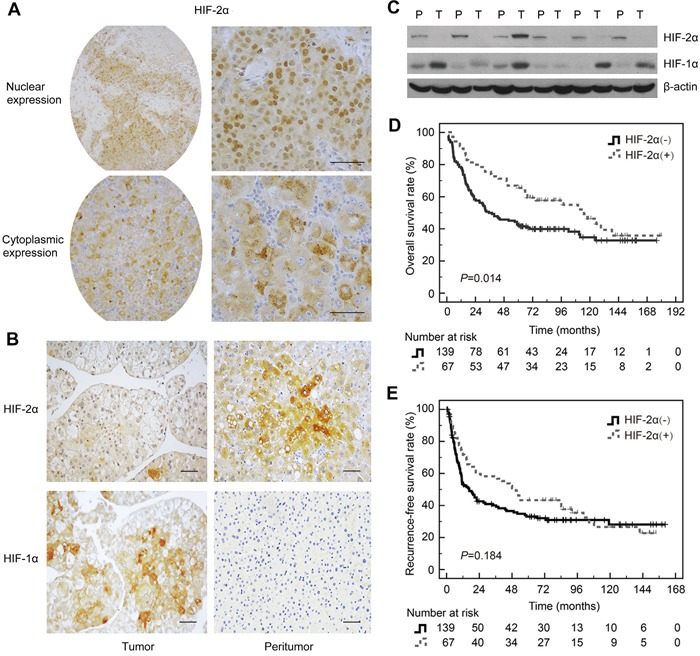
Expression of HIF-2α in HCC tissues and the correlation with prognosis **A.** Immunohistochemical detection of HIF-2α in the nucleus and cytoplasm of HCC. Anti-HIF-2α monoclonal antibody (190b) was purchased from Santa Cruz. **B.** Representative images of HIF-2α and HIF-1α staining in HCC tissues and the corresponding peritumoral tissues. Scale bar, 10mm. Anti-HIF-2α monoclonal antibody (190b) was provided by Santa Cruz. **C.** HIF-2α and HIF-1α protein levels in HCC tissues and the paired corresponding tissues were verified by Western blot. β-actin was used as a control. **D.** Kaplan-Meier analysis of overall survival (OS) in 206 HCC patients based on HIF-2α expression. **E.** Kaplan-Meier analysis of recurrence-free survival (RFS) in 206 HCC patients based on HIF-2α expression.

To investigate the role of HIF-2α in the development of HCC, we first determined the levels of HIF-2α in tumor and paired peritumoral tissues. HIF-2α was localized mainly in the cytoplasm of the cells in both tumor tissues and peritumoral tissues (Figure 1A). Unlike HIF-1α, HIF-2α protein was significantly lower in tumor than in peritumoral tissues (Figure 1B and 1C). To detect HIF-2α, we used two types of anti-HIF-2α antibodies. One was clone 190b, which is a monoclonal antibody raised against recombinant HIF-2α of human origin. The other one was a polyclonal antibody generated from the peptide corresponding to mouse HIF-2α aa 632-646. Both are suitable for HIF-2α detection in human samples [14, 15]. The results consistently showed that the level of HIF-2α was higher in peritumoral tissues than in HCC tissues no matter which primary antibody was used (Supplementary Figure S1).

We further investigated the relationship between the level of HIF-2α and the clinicopathological features. As shown in Supplementary Table S2, the level of HIF-2α in large size (> 5 cm) tumors was significantly lower than that in small size tumors (≤ 5 cm) (*P*<0.001). However, no correlation was found between HIF-2α and other clinicopathological features including age, gender, cirrhosis, serum AFP level, tumor differentiation grade and macroscopic vascular invasion. Kaplan-Meier analysis showed that patients with high HIF-2α levels in HCC tissues had significantly longer OS than those with low levels (Figure 1D). The mean overall survival time of patients with high HIF-2α levels in tumor tissues was 103.2 ± 8.9 months, while it was only 75.3 ± 6.5 months in patients with low HIF-2α levels (*P*=0.014). Meanwhile, in univariate analysis, the high level of HIF-2α had an impact on mortality (hazard ratio [HR] =0.615, 95% CI: 0.410-921, *P*=0.018) (Supplementary Table S3). Furthermore, HIF-2α levels and other clinicopathlogical variables including gender, age, tumor diameter, number of tumor lesions, presence of liver cirrhosis, preoperative serum alanine aminotransferase, bilirubin, AFP and albumin level, histologic differentiation, and macroscopic vascular invasion were included in a multivariable model using Cox regression. After adjustment for co-variables, the corresponding values were 0.734 (0.459 to 1.174) (*P*=0.197) (Supplementary Table S3), indicating that the level of HIF-2α protein could not be considered an independent predictor for overall survival of HCC patients. Kaplan-Meier and Cox Proportional Hazards regression analyses demonstrated that HIF-2α protein level was not associated with RFS of HCC patients (Figure 1E and Supplementary Table S4).

### HIF-2α inhibits HCC cell growth mainly through inducing apoptosis

**Figure 2:**
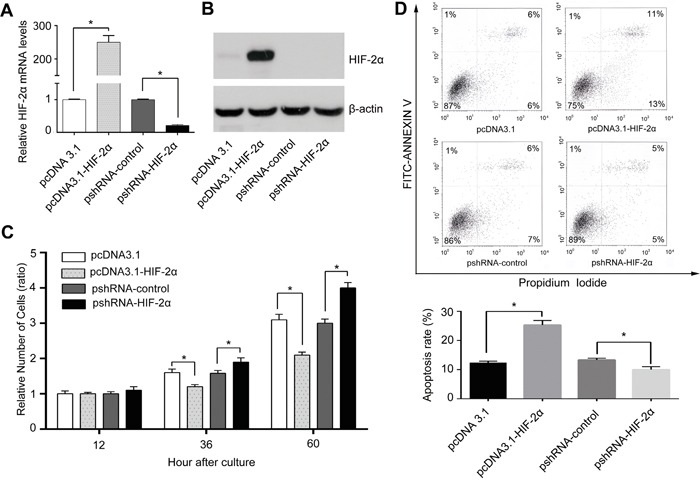
HIF-2α inhibited HCC cell proliferation mainly through inducing apoptosis *in vitro* HepG2 cells were transient transfected with pcDNA3.1-HIF-2α, or pshRNA-HIF-2α recombinant vector or corresponding control plasmid (pcDNA3.1, pshRNA-control). **A.** Real-time PCR analyzed the mRNA levels of HIF-2α in each group, error bars represented standard deviation (n=3), **P<0.01. **B.** Protein levels of HIF-2α and **C.** proliferative ability of cells with different levels of HIF-2α. The relative absorbance at each time point (12h, 36h, 60h) was normalized to that detected at the first time point (12h), *P<0.05 and **P<0.01. **D.** Flow cytometry with PI/Annexin V FITC double staining was used to detect apoptosis. Column bars indicated mean apoptotic percentage, error bars represented standard deviation, *P<0.05 and **P<0.01.

To uncover the mechanisms of HIF-2α leading to favourable outcome in HCC patients, we manipulated the level of HIF-2α in liver cancer cells by the over-expression (transfected with the HIF-2α expression plasmid pcDNA3.1-HIF2a) and the downregulation (HIF-2α shRNA) (Figure 2A and 2B). MTT assay showed that under normoxic conditions the elevated level of HIF-2α in HepG2 cells was associated with a slower growth rate, compared to the respective control, whereas cells transfected with HIF-2α shRNA grew faster than the control (Figure 2C). Next, we investigated whether the altered growth rate was the result of changes in the cell death rate. To this end, propidium iodide and annexin-V was applied to determine the proportion of apoptotic cells in the HIF-2α over-expressing cells. We found that 25.0±1.5% cells were apoptotic in the high HIF-2α group while there was only 12.0±0.6% in the control group (Figure 2D). Similar findings were also obtained in another HCC cells (Supplementary Figure S2). The results of HIF-2α overexpression by pcDNA3.1-HIF2a on cell growth and apoptosis were confirmed using HIF-2α lentiviral vector-mediated HIF-2α overexpression (data not shown).

### HIF-2α induces apoptosis through up-regulating the expression of Bak, ZBP-89 and PDCD4 in HepG2 and Hep3B cells

**Figure 3:**
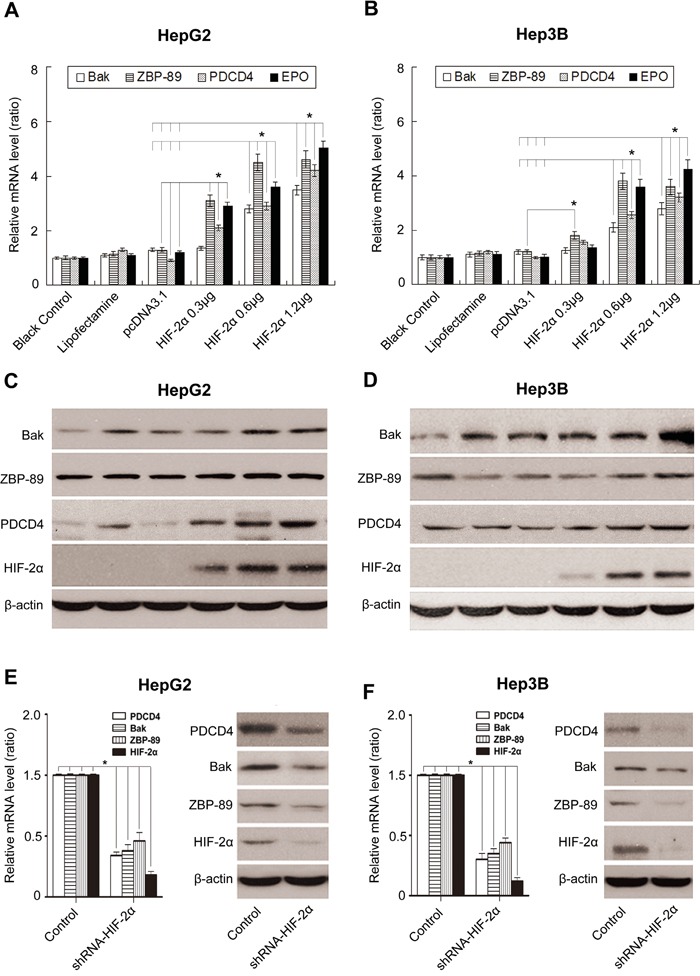
Effects of HIF-2α on the expression of Bak, ZBP-89, PDCD4 and erythropoietin (EPO) in HCC cells Real-time PCR **A., B.** and Western blotting **C., D.** analysis of the expression of Bak, ZBP-89, PDCD4 and EPO in HepG2 and Hep3B cells (respectively transfected with 0, 0.3, 0.6 and 1.2 μg pcDNA3.1-HIF-2α for 24 h). Real-time PCR and Western blotting analyses of Bak, ZBP-89 and PDCD4 levels in HepG2 **E.** and Hep3B **F.** cells treated with HIF-2α shRNA. β-actin was used as a control. Error bars represented standard deviation (n=3), **P<0.01.

**Figure 4:**
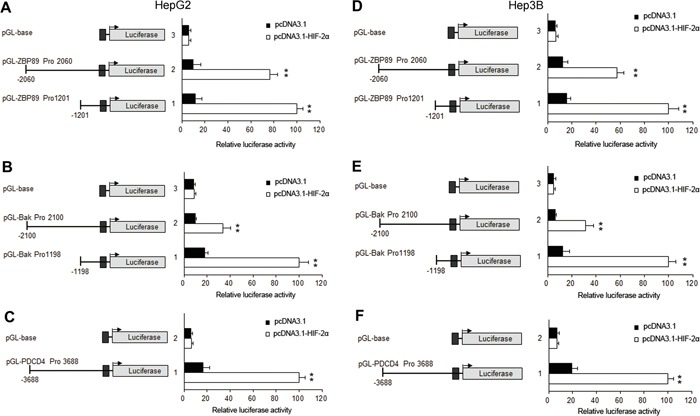
ZBP-89, Bak and PDCD4 promoter activities were significantly increased after HIF-2α transfection in HepG2 and Hep3B cells HepG2 and Hep3B cells were transiently transfected with 0.4 μg of individual (ZBP-89 or Bak or PDCD4) promoter luciferase construct, pGL3 Basic or pGL3 control plasmids, together with 10 ng Renilla luciferase plasmid which served as an inner control for transfection efficiency and 0.8 μg pcDNA3.1 or pcDNA3.1- HIF-2α. The cells were lysed to determine the relative firefly luciferase activity, and the change of the activity was calculated in fold. The results were the mean of 3 independent experiments, and the values are expressed as mean ±SD (**P<0.01).

**Figure 5:**
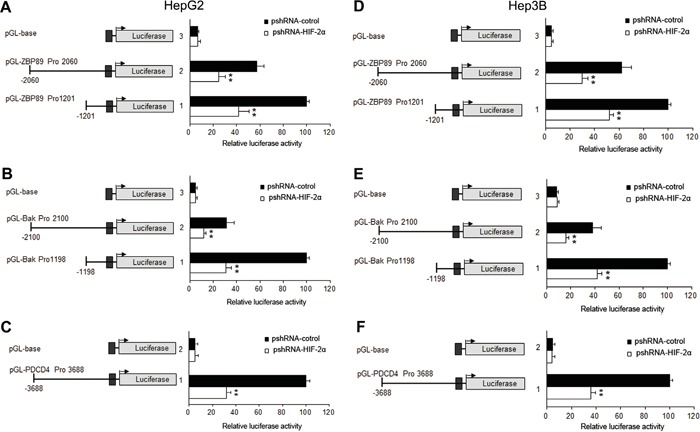
Effects of HIF-2α shRNA on the promoter activities of PDCD4, Bak, ZBP-89 in HepG2 and Hep3B cells HepG2 and Hep3B cells were transiently transfected with 0.4 μg of individual (ZBP-89 or Bak or PDCD4) promoter luciferase construct, pGL3 Basic or pGL3 control plasmids, together with 10 ng Renilla luciferase plasmid which served as an inner control for transfection efficiency and 0.8 μg pshRNA-control or pshRNA-HIF-2α. The cells were lysed to determinate the relative firefly luciferase activity and the change of the activity was calculated in fold. The results were the mean of 3 independent experiments, and the values are expressed as mean ±SD (**P<0.01).

To further reveal the underlying mechanisms of HIF-2α-mediated apoptosis, we investigated the impact of HIF-2α on the three apoptotic proteins Bak, ZBP-89 and PDCD4 under normoxia conditions. Our results showed that the over-expression of HIF-2α could not only stimulate the expression of its target gene erythropoietin (EPO), but also the expression of Bak, ZBP-89 and PDCD4. Under hypoxic conditions, the inhibition of HIF-2α could decrease the levels of ZBP-89, Bak and PDCD4 (Figure 3 and Supplementary Figure S3). Meanwhile, luciferase assay showed that the promoter activities of Bak, ZBP-89 and PDCD4 were significantly increased after pcDNA-HIF-2α transfection but were obviously decreased after pshRNA-HIF-2α transfection (Figure 4 and (Figure 5). These findings are in line with the result that the promoter regions of Bak, ZBP-89 and PDCD4 genes contain several potential HIF-2α binding sites (Supplementary Figure S4). Taken together, these data have demonstrated that ZBP-89, Bak and PDCD4 may be novel target genes of HIF-2α and that they likely contribute to HIF-2α-mediated apoptosis of HCC cells.

### Over-expression of HIF-2α induces apoptosis in mouse HepG2-cell xenografts

**Figure 6:**
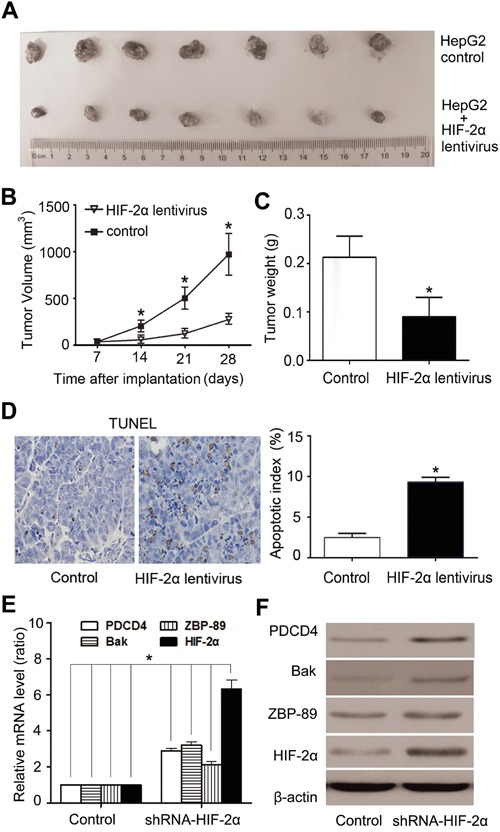
Over-expression of HIF-2α induced HCC growth arrest and high apoptosis rate in HepG2-cell xenografts in nude mice **A.** Tumors formed by implanted cells with different expression levels of HIF-2α. **B.** Tumor volume. Tumor volumes were calculated and data were plotted using the geometric mean for each group vs. time. Each point represents the mean tumor volume (± sd) of measurements from the 7 mice in each treatment group. **P<0.01, two-tailed test. **C.** The weight of tumors was significantly lower in HIF-2α lentivirus-infected group than the control group. **P<0.01, two-tailed test. **D.** Cells positive for TUNEL staining were statistically increased in HIF-2α-over-expressing tumors, compared with the control (**P < 0.01, Student's t test). Real-time PCR **E.** and western blotting analysis **F.** of HIF-2α, ZBP-89, Bak, PDCD4 expression in xenograft tumors after mouse sacrifice.

Mouse HepG2-cell xenografts were set up to verify the effect of HIF-2α on tumor growth and apoptosis *in vivo*. As shown in Figure 6A, tumors in the control group reached 944.3 ± 82.1mm3 in 4 weeks, while tumors formed by HIF-2α lentivirus-infected cells reached only 271.4± 153.5 mm^3^ (Figure 6B). There was also a significant difference in tumor weight between these two groups. The tumor weight of HIF-2α lentivirus group was much lower than that of control group (Figure 6C). TUNEL staining showed that HIF-2α-lentivirus infection group had more apoptotic cells than the control group. Quantification of TUNEL-stained samples showed a 3- to 4-fold increase (*P*<0.05) in the number of TUNEL-positive cells in HIF-2α lentivirus groups, compared with the control (Figure 6D). To investigate the effect of HIF-2α on the expression of Bak, ZBP-89 and PDCD4 *in vivo*, we detected the expression of HIF-2α, Bak, ZBP-89 and PDCD4 in xenograft tumor tissues by real-time PCR, western blotting and immunohistochemical staining analysis. The results showed that, as compared to controls, the expression of Bak, ZBP-89 and PDCD4 was dramatically increased in tumor cells over-expressed HIF-2α (Figure 6E, 6F and Supplementary Figure S5).

## DISCUSSION

There are limited reports on HIF-2α in HCC with contradictory conclusions regarding HIF-2α function [14, 15]. Bangoura *et al.* suggested that HIF-2α played a positive role in promoting HCC because the high level of HIF-2α was associated with tumor angiogenesis and a poor outcome [14], whereas Sun *et al.* found that the high level of HIF-2α protein was correlated with a good outcome in patients with HCC and that the expression of pro-apoptotic proteins was much more higher in HCC patients with high HIF-2α than those with low HIF-2α [15]. Therefore, the role of HIF-2α in HCC remains unclear. We analyzed the level of HIF-2α protein in HCC tissues and paired peritumoral tissues. Interestingly, the level of HIF-2α in tumor tissues was found to be significantly lower than in peritumoral tissues, while the level of HIF-1α showed the opposite result, higher in tumor tissues than in paracancerous tissues. Our HIF-2α results were consistent with Sun *et al.* [15]. It should note that the primary anti-HIF-2α antibody used in the Bangoura and Sun reports were different. Bangoura *et al.* used mouse anti-HIF-2α (190b) monoclonal antibody (raised against recombinant human HIF-2α) from Santa Cruz, while Sun *et al.* used rabbit anti-HIF-2α polyclonal antibody (ab199) from Abcam (corresponding to amino acids 632-646 of HIF-2α) [14, 15]. These two antibodies may target different regions of HIF-2α protein. To ascertain our findings as well as to rule out the possibility that different antibodies may produce the opposite results, we examined our samples by both above anti-HIF-2α antibodies and consistent results were obtained. Therefore, we conclude that the different antibodies used are not responsible for the inconsistent results showed in the previous reports [14, 15]. Both immunohistochemical staining and Western blotting results suggest that the level of HIF-2α is down-regulated in HCC tissues compared with peritumoral tissues.

After examining the level of HIF-2α in HCC, we analyzed the relationship between the level of HIF-2α and clinicopathological features of HCC. We found that HIF-2α level in HCC tissues was negatively correlated with tumor size but positively with OS rate in HCC patients. Meanwhile, we explored the influence of HIF-2α protein on HCC cell proliferation and apoptosis. Our results demonstrated that the over-expression of HIF-2α induced HCC cell growth arrest and apoptosis, whereas the knockdown of HIF-2α expression increased cell viability and decreased apoptosis of HCC cells. These results together with HIF-2α expression data suggest that HIF-2α may function as an anti-tumor molecule in HCC. In fact, the pro-apoptotic function of HIF-2α has also been found in other cancers such as glioblastoma and renal carcinoma [21,22].

To understand the molecular mechanisms by which HIF-2α inhibited cell growth and induced apoptosis, we explored the influence of HIF-2α on the three pro-apoptotic proteins Bak, ZBP-89 and PDCD4 [23, 24]. We used EPO as a positive control and found that HIF-2α could enhanced not only the expression of EPO, but also the expression of Bak, ZBP-89 and PDCD4 at both mRNA and protein levels. Moreover, several potential HIF-2α binding sites were discovered in the promoter regions of Bak, ZBP-89 and PDCD4 genes, and HIF-2α could increase the activity of these promoters. Both ZBP-89 and PDCD4 are known to promote apoptosis of HCC cells [23,25]. Therefore, we conclude that HIF-2α-mediated induction of apoptosis is likely through stimulating the expression of ZBP-89, PDCD4 and Bak in HCC.

This study had some limitations. Firstly, the sample size was not large which may reduce the power of statistical analysis. Secondly, our current study was retrospective in nature. However, the robust inclusion criteria (only the patients who received curative hepatectomy, were positive for HBV and continuously followed up for at least 5 years were enrolled) allowed recruitment of highly homogeneous patients and consequently enhanced quality of evidences in the present study. Third, all of HCC patients studied are with HBV infection. Thus our data may not be applicable for non-HBV-related HCC.

In conclusion, our findings have demonstrated the low expression patterns and pro-apoptotic effect of HIF-2α in HCC, suggesting that the upregulation of HIF-2α may benefit HCC patients. It should note that HIF-2α is positively associated with OS but not DFS. Though the upregulation of HIF-2α may inhibit the growth of HCC, HIF-2α itself may not be a predictive factor for HCC recurrence.

## MATERIALS AND METHODS

### Patients and clinicopathologic information

206 patients with HCC who underwent an operation between July 1999 and December 2009 at the Prince of Wales Hospital, Hong Kong were recruited, and they all met the following criteria. 1) newly diagnosed HCC patients with liver function tests of Child-Pugh grade A; 2) the patient underwent radical resection; 3) patients without history of anti-cancer therapy; 4) patients without any distant metastasis; 5) patients without other types of malignant tumors, autoimmune, liver disease or serious heart, lung, kidney, or blood diseases; 6) seropositive for hepatitis B surface antigen and seronegative for HCV; 7) information for follow-up was available and continuously followed up for at least 5 years. All patients or their legal representative signed an informed consent for participation in this study. Human studies were approved by the Joint CUHK-NTEC Clinical Research Ethics committee. Complete removal of cancer tissues with no evidence of residual microscopic tumor cells was defined as curative resection. Patients were confirmed without distant metastasis prior to surgery by axial imaging consisting of either 3-phase computerized tomography (CT) with intravenous contrast or multiphase magnetic resonance imaging (MRI) with intravenous contrast. By reviewing medical records, we recorded the following information: serum albumin, alanine aminotransferase, bilirubin and AFP levels. All liver specimens were examined by pathologists who were blinded to all patient identities and clinical outcomes.

### Follow-up evaluations

The plan for follow-up evaluations was: (1) every three months in the first postoperative year; (2) every four months in the second postoperative year; and (3) every six months thereafter. CT or MRI images were used to diagnose tumor recurrence during during follow-up. Death of patients was obtained from the social security death index, medical records, or notifications from family of the deceased. The primary end point was mortality or tumor recurrence. OS was defined as the interval from the date of operation to the death or 26 June 2014; RFS was defined as time from curative hepatectomy to the first occurrence.

### Cell culture and transfection

The human HCC cell line HepG2 was purchased from American Type Culture Collection (Rockville, MD). The human HCC cell lines (Hep3B, and Bel-7404) were obtained from the Institute of Cell Biology (SIBS, CAS, Shanghai, China). HepG2, Bel-7404 and Hep3B cells were maintained in Dulbecco's modified Eagle medium (Invitrogen, Carlsbad, CA). For normoxic conditions, the cells were cultured in the atmosphere with 5% CO2 at 37°C. For mimicking the hypoxic conditions, the cells were cultured in an atmosphere with 1% O2, 5% CO2, and 94% N2 at 37°C. Cell transfection were performed as previously reported [26].

### Reagents

Antibodies against HIF-1α and β-actin obtained from Santa Cruz Biotechnology (Santa Cruz, CA). Anti-HIF-2α polyclonal antibody (ChIP Grade ab199) was provided by Abcam (Cambridge, MA). Mouse anti-HIF-2α/EPAS1 monoclonal antibody (190b) was purchased from Santa Cruz Biotechnology.

### Immunohistochemical analysis

Immunohistochemical analysis was performed as described previously [27, 28]. The levels of HIF-2α proteins were scored according to the number of cells exhibiting the cytoplasmic and nuclear staining using the same classification system as Sun *et al*'s: nuclear or cytoplasm staining < 50% of tumor cells was considered low expression, while the remaining cases were considered as high expression [15].

### Real-time PCR assay and western blot

RNA extraction, cDNA synthesis, quantitative real-time PCR (qRT-PCR) reactions, and Western blot were performed as before [27]. The primer sequences can be found in the Supporting Materials and Methods (Supplementary Table S5). β-actin was applied as an internal standard control in both PCR and Western blot.

### Apoptosis analysis

To quantify the extent of apoptosis, cells were harvested with trypsin without EDTA, and visualized by Annexin V-FITC/propidium iodide staining according to the manufacturer's protocol (Invitrogen, Carlsbad, CA).

### Firefly luciferase reporter constructs and luciferase assays

Human ZBP-89 and Bak promoters were constructed using PCR cloning from human genomic DNA. The detailed procedures were described in the Supporting Materials and Methods. Transient transfection and luciferase assays were performed as described previously [27].

### Xenograft animal model

All animal procedures were approved by the Animal Experimentation Ethics Committee of the Chinese University of Hong Kong and are in accordance with the Department of Health (Hong Kong) guidelines in Care and Use of Animals. The detailed procedures are described in the Supporting Materials and Methods.

### Statistical analysis

Continuous data and discrete variables were expressed as the median and range or absolute values with relative frequencies, respectively. Categorical variables were analyzed using Chi-square or Fisher's exact tests (if any expected frequency is less than 1 or 20% of expected frequencies are less than or equal to 5), and continuous variables were analyzed using the nonparametric Wilcoxon rank-sum test. The Kaplan–Meier method followed by comparison using log-rank tests was used to calculate the OS and RFS rates. Univariable or multivariable Cox proportional hazard regression was performed to evaluate the predictive values of HIF-2α and other clinicopathologic features (age, gender, tumor diameter, number of tumor lesions, presence of liver cirrhosis, preoperative serum alanine aminotransferase, bilirubin, and albumin level, histologic differentiation and macroscopic vascular invasion). The cut-off points for serum ALT, albumin, bilirubin and AFP were 80 IU/L, 35 g/L, 20 μmol/L and 400 ng/ml, respectively, according to previous experience [29, 30]. The final model was selected by backward elimination. Statistical analysis was performed using SPSS version 16.0 (SPSS, Chicago, IL) and STATA version 12.0 (StataCorp LP, College Station, TX). A two-tailed p-value less than 0.05 was considered statistically significant. Multiple imputation methods were used to estimate the missing values. Iterative rounds of imputation (N=25) were performed using the mi command in STATA version 12.0.

## SUPPLEMENTARY MATERIALS FIGURES AND TABLES


