# Supplementary material for: Downregulation and pro-apoptotic effect of hypoxia-inducible factor 2 alpha in hepatocellular carcinoma
Source: Oncotarget. 2016 Apr 23;7(23):34571–81. doi: 10.18632/oncotarget.8952 (PMC5085177; doi:10.18632/oncotarget.8952)
Supplement: Supplementary file 1 [file oncotarget-07-34571-s001.pdf]

Downregulation and pro-apoptotic effect of hypoxia-inducible factor 2 alpha in hepatocellular carcinoma

SUPPLEMENTARY MATERIALS AND METHODS

Cell lines and cell cultures

The human HCC cell line HepG2 was purchased from American Type Culture Collection (Rockville, MD). The human HCC cell lines (Hep3B, and Bel-7404) were obtained from the Institute of Cell Biology (SIBS, CAS, Shanghai, China). HepG2, Bel-7404 and Hep3B cells were maintained in Dulbecco's modified Eagle medium (Invitrogen, Carlsbad, CA), supplemented with 10% heat-inactivated fetal bovine serum. For normal condition, the cell was cultured in the atmosphere with 5% CO2 at 37°C. For mimicking the hypoxic conditions, the cell was cultured in an atmosphere with 1% O2, 5% CO2, and 94% N2 at 37°C (Incubator: Galaxy R incubator, Model No: 170-300, RS Biotech, UK).

Plasmids

The pcDNA3.1-HIF-2α, shRNA-HIF-2α plasmid and lentiviral gene expression vector pGV287-HIF-2α, lentiviral packaging vector pHelper 1.0 and pHelper 2.0 were purchased from Shanghai GeneChem Co., Ltd. (Shanghai, China). PDCD4 promoter (-3688/+205) was obtained from -Professor Jorg Hendrik LEUPOLD (Department of Experimental Surgery, Medical Faculty Mannheim, University of Heidelberg, Germany) as a kind gift.

MTT assay

Cell proliferation was determined by MTT assay. Approximately 1×10^4 cells were seeded in 96-well microculture plates and incubated overnight. In the second day, different treatments were carried out and incubated for 0 to 4 days depended on the specific experiment. After incubation, chemical MTT 20 µl (5 mg/ml) was added to each well to the final concentration of 50 µg/ml, and the cells were further incubated for 4 h at 37 °C. At the end of the incubation, remove supernatant carefully without disturb the cells in the bottom and added 200 µl DMSO into each well. Shake the whole plate for 15 min. The microtiter plate was spectrophotometrically read at a wavelength of 570 nm, with a reference wavelength of 630 in a multi-well plate reader (Bio-Rad, Hercules, CA). Cell proliferation rate (%) = (Absorbance of treated cell/ Absorbance of untreated cell) ×100%.

Firefly luciferase reporter constructs and luciferase assays

Two different ZBP-89 (-2060–60 and -1201–60 relative to the transcription start site of NM_021964) and Bak (-2100–33 and -1198–33 relative to the transcription start site of NM_001188.3) promoter fragments were amplified from genomic DNA of human HCC cells by polymerase chain reaction (PCR). The PCR reaction was carried out at 94°C for 3 min, followed by 35 cycles at 95°C for 30 sec, 52-56°C for 45 sec and 72°C for 1 min. The PCR fragments of four promoter were inserted into luciferase reporter vector pGL3-Basic (Promega, Madison, WI, USA) digested by *Sac*I/*Xho*I (Takara Biotechnology Co., Ltd., Dalian, China). All constructs were confirmed by *Sac*I/*Xho*I digestion and DNA sequencing was confirmed by sequencing (ABI PRISMTM, Applied Biosystems, Foster City, CA). HCC cells (HepG2, Hep3B, and Bel-7404) were seeded into 24-well plates ($1\text{x}10^5$ cells/well). After 48 h, the cells were co-transfected with promoter plasmid (Bak or ZBP-89 or PDCD4 or pGL3-Basic), pcDNA3.1 or pcDNA3.1-HIF-2α, pRL-TK Renilla Luciferase Reporter (Promega), pshRNA-HIF-2α or a negative control with aid of Lipofectamine 2000 (Invitrogen) according to the manufacturers' instructions. At 24 h after the transfection, the cells were washed twice with phosphate-buffered saline (PBS) and then were harvested and lysed in RIPA. The luciferase assays were performed with the Luciferase Assay System (Promega). The following primers were used for ZBP-89 and Bak promoter construction.

ZBP-89 pro_SacI-F1: GGT GAGCTCTGAATATG CGGTTAACTGTG

Z BP-89 pro_SacI-F2: GAAGAGCTCAGGCATC ACGATTTACTAAG

Z BP-89 pro_XhoI-R: GTTCTCGAGCCACTGCC GGCCCGGTGCA

Bak1 pro_SacI-F1: CGAGAGCTCAGTTTCGCTCT TGTTGACC

Bak1 pro_SacI-F2: CAAGAGCTCGGAACTCCT GCACCTGGC

Bak1 pro_XhoI-R: GAACTCGAGGAAGTTGCT CTGTGGC.

Xenograft animal model

Female nude mice (4 weeks old) were purchased from the Laboratory Animal Services Centre of the Chinese University of Hong Kong (Hong Kong SAR, China). The animals were kept in a temperature controlled room (23 ± 2°C) with a 12 h light: 12 h dark cycle. To generate HCC xenografts, Lv-HIF-2α or Lv-control-infected HepG2 cells were harvested from mid-log phase cultures using trypsin-EDTA, pelleted and resuspended in phosphate buffer saline (PBS) to a final cell count of 5×10^6/ml. A volume of 150 µl of the cell suspension was injected subcutaneously in the left flank of each mouse. Tumor weight was calculated weekly using the equation: $(\text{length} \times (\text{width})^2)/2$. Four weeks after the cell implantation, the tumors were excised intact and weighted. Tumor tissues were subjected to H&E staining and immunohistochemical staining. The tissue lysate was analyzed by real-time PCR and Western blot to detect the expression of HIF-2α, ZBP-89, and PDCD4.

Supplementary Table S1: Main demograhic, biochemical and clinical characteristics of the 206 HCC patients

Variable	Unit	Value
Age	years	57.2 (31-84)
Gender	male	177 (85.9)
Albumin	g/L	38.1 (29-46)
ALT	U/L	50.0 (11-227)
Total bilirubin	g/L	10.3 (3-20)
HCC diameter	cm	5.2(1.1-15)
AFP	ng/ml	83 (2-699800)

Data are shown as median value (range) or absolute frequency (%). ALT, alanine aminotransferase; HCC, hepatocellular carcinoma.

Supplementary Table S2: Correlations of HIF-2α protein expression in surgical specimens of HCC with clinicopathological characteristics

Parameters	HIF-2α#		*P*
	Low (n=139)	High (n=67)	
Age			0.958
≤50y	41	20	
>50y	98	47	
Gender			0.808
Male	19	10	
Female	120	57	
Cirrhosis			0.278
Absence	50	19	
Presence	89	48	
Tumor size			0.000*
≤5cm	62	53	
>5cm	77	14	
AFP			0.419
≤400μg/L	87	38	
>400μg/L	52	29	
Number of tumor			0.982
Single	106	51	
muti-tumor	33	16	
Histological grade			0.374
Well	15	11	
Moderate	105	50	
Poor	19	6	
Macroscopic vascular invasion			0.707
Absence	101	47	
Presence	38	20	

Please refer to Immunohistochemical analysis Section for the definition of low or high expression of HIF-2α. * indicated $P<0.05$.

Supplementary Table S3: Cox proportional Hazard regression analysis of patients' overall survival

	Univariable		Multivariable	
**Variables**	**HR (95% CI)**	***P***	**HR (95% CI)**	***P***
Gender (male vs female)	0.734 (0.412-1.308)	0.295	0.475 (0.063-3.581)	0.470
Age (> 50 vs ≤ 50)	1.629 (1.096-2.420)	0.016	1.267 (0.824-1.947)	0.280
Number of tumor lesions (multiple vs single)	2.283 (1.543-3.378)	0.000	**2.083 (1.373-3.165)**	**0.001**
Macroscopic vascular invasion (present vs absent)	1.833 (1.249-2.690)	0.002	**1.941 (1.341-2.810)**	**0.000**
ALT (> 80 IU/L vs ≤ 80 IU/L)	1.117 (0.691-1.806)	0.652	1.060 (0.612-1.695)	0.943
Albumin (> 35 g/L vs ≤ 35 g/L)	0.959 (0.568-1.618)	0.876	0.957 (0.544-1.684)	0.879
Bilirubin (> 20 μmol/L vs ≤ 20 μmol/L)	1.112 (0.713-1.736)	0.639	1.123 (0.687-1.835)	0.642
AFP (> 400 ng/ml vs ≤ 400 ng/ml)	1.608 (1.124-2.300)	0.009	**1.634 (1.135-2.352)**	**0.008**
Cirrhosis (present vs absent)	1.713 (1.136-2.585)	0.010	**1.642 (1.033-2.611)**	**0.036**
Histological differentiation (moderately vs well)	1.146 (0.674-1.948)	0.614	1.004 (0.543-1.858)	0.989
Histological differentiation (poorly vs well)	1.593 (0.840-3.020)	0.153	1.600 (0.759-3.375)	0.217
Greatest tumor diameter (> 5 cm vs ≤ 5 cm)	2.075 (1.435-3.002)	0.000	**2.196 (1.233-2.778)**	**0.003**
HIF-2α (high vs low)	0.615 (0.410-0.921)	0.018	0.734 (0.459-1.174)	0.197

Supplementary Table S4: Cox proportional Hazard regression analysis of patients' recurrence-free survival

	Univariable		Multivariable	
**Variables**	**HR (95% CI)**	**P**	**HR (95% CI)**	**P**
Gender (male vs female)	1.010 (0.605-1.686)	0.970	1.968 (0.682-5.674)	0.210
Age (> 50 vs ≤ 50)	1.427 (0.956-2.132)	0.082	1.333 (0.846-2.100)	0.215
Number of tumor lesions (multiple vs single)	3.279 (2.193-4.902)	0.000	**3.436 (2.293-5.154)**	**0.000**
Macroscopic vascular invasion (present vs absent)	2.022 (1.397-2.927)	0.000	**2.660 (1.733-4.082)**	**0.000**
ALT (> 80 IU/L vs ≤ 80 IU/L)	1.049 (0.670-1.641)	0.835	1.316 (0.860-2.013)	0.206
Albumin (> 35 g/L vs ≤ 35 g/L)	0.972 (0.659-1.435)	0.888	0.924 (0.486-1.972)	0.809
Bilirubin (> 20 μmol/L vs ≤ 20 μmol/L)	1.036 (0.679-1.580)	0.870	1.318 (0.843-2.060)	0.226
AFP (> 400 ng/ml vs ≤ 400 ng/ml)	1.752 (1.223-2.510)	0.002	**1.676 (1.161-2.419)**	**0.006**
Cirrhosis (present vs absent)	1.640 (1.102-2.442)	0.015	**1.623 (1.022-2.635)**	**0.040**
Histological differentiation (moderately vs well)	1.164 (0.651-2.079)	0.607	0.817 (0.473-1.392)	0.446
Histological differentiation (poorly vs well)	1.701 (0.726-3.984)	0.221	1.262 (0.641-2.456)	0.511
Greatest tumor diameter (> 5 cm vs ≤ 5 cm)	1.937 (1.357-2.766)	0.000	**2.092 (1.381-3.175)**	**0.001**
HIF-2α (high vs low)	1.261 (0.870-1.828)	0.222	1.236 (0.799-1.912)	0.340

Supplementary Table S5: Real-time PCR primer sequences

Gene	Primer	Accession No.	Product size (bp)
HIF-2α	5'-TCATGCGACTGGCAATCAGC-3'	NM_001430.4	142
	5'-GTCACCACGGCAATGAAACC-3'		
ZBP-89	5'-TAAATGTGGCGGCATAGACG-3'	NM_021964	131
	5'-CCTGGTGAGG CATACTTCG-3'		
Bak	5'-AGGAACAGGAGGCTGAAGGG-3'	NM_001188.3	135
	5'-CATAGCGTCGGTTGATGTCG-3'		
PDCD4	5'-TGTGCCAACCAGTCCAAA-3'	NM_145341.3	167
	5'-CATCATCATAGTTAGGATCTTTCAC-3'		
EPO	5'-ATATCACTGTCCCAGACACCAA-3'	NM_000799	190
	5'-AGAGTGGTGAGGCTGCGAAGGCCAC-3'		
β-actin	5'- AGTTGCGTTACACCCTTTCTTGAC-3'	NM_001101	167
	5'- GCTCGCTCCAACCGACTGC-3'		

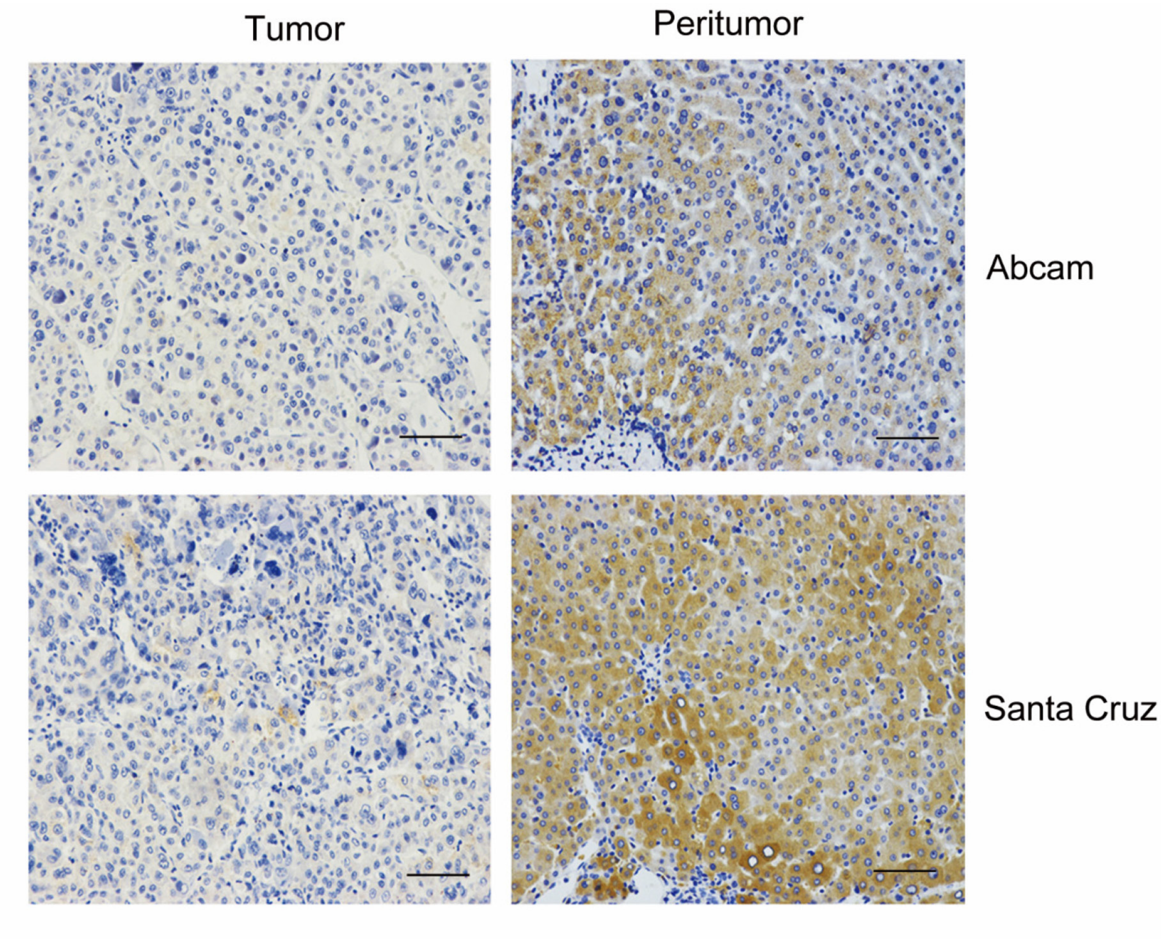

Supplementary Figure S1: Immunohistochemical staining of HIF-2α in in HCC tissues and the corresponding peritumoral tissues using two different anti-HIF-2α antibodies (×200). Anti-HIF-2α polyclonal antibody (ChIP Grade ab199) was provided by Abcam. Mouse anti-HIF-2α/EPAS1 monoclonal antibody (190b) was purchased from Santa Cruz Biotechnology. Scale bar, 10mm.

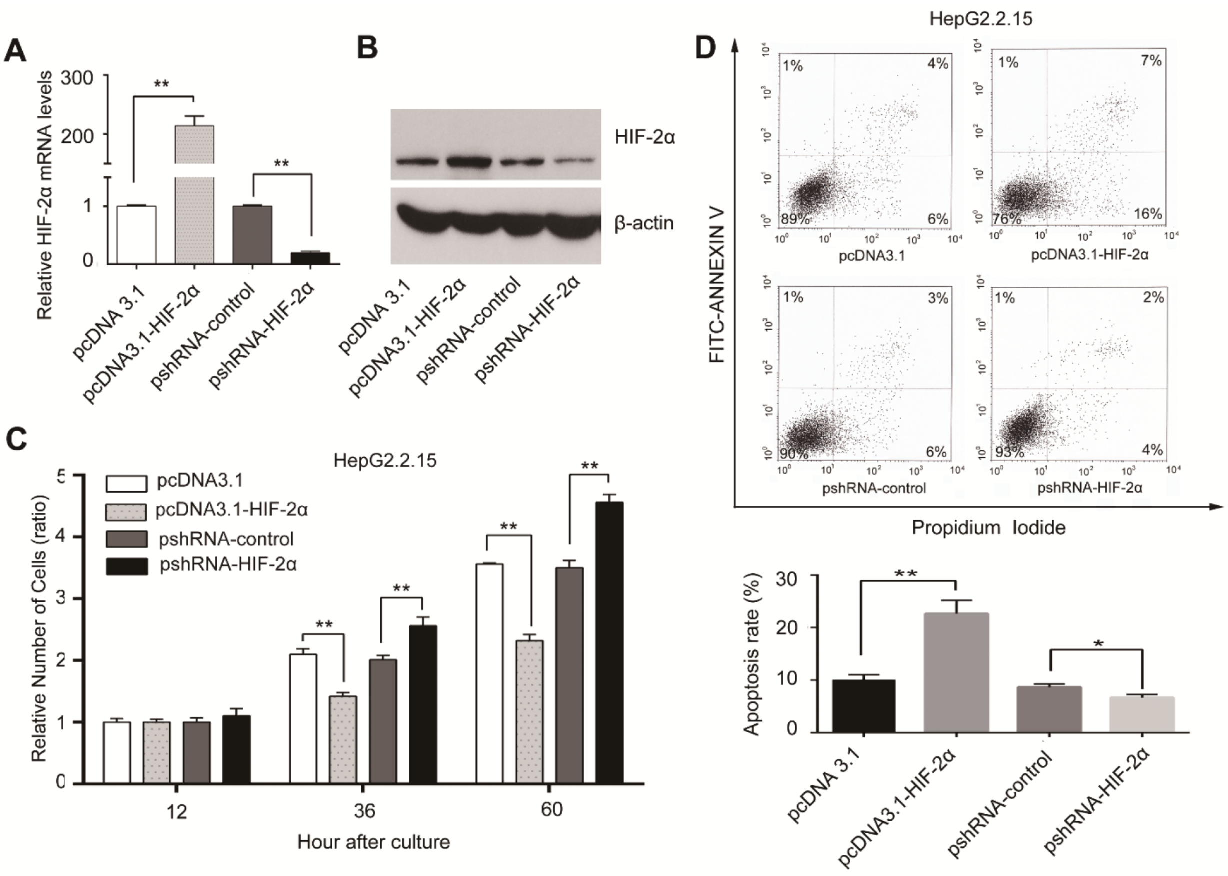

Supplementary Figure S2: Confirmation of HIF-2α-mediated inhibition of HCC cell proliferation mainly through inducing apoptosis in HepG2.2.15 cells. Cells were transiently transfected with pcDNA3.1-HIF-2α or pshRNA-HIF-2α recombinant vector or corresponding control plasmid (pcDNA3.1, pshRNA-control). **A.** Real-time PCR analysis of the expression of HIF-2α in each group, error bars represented standard deviation (n=3), **P*<0.05. **B.** Protein levels of HIF-2α and **C.** proliferative ability of cells with different levels of HIF-2α. The relative absorbance at each time point (12h, 36h, 60h) was normalized to that detected at the first time point (12h), **P*<0.05. **D.** Flow cytometry with PI/Annexin V FITC double staining was used to detect apoptosis. Column bar indicated mean apoptotic percentage, error bars represented standard deviation, *P<0.05.

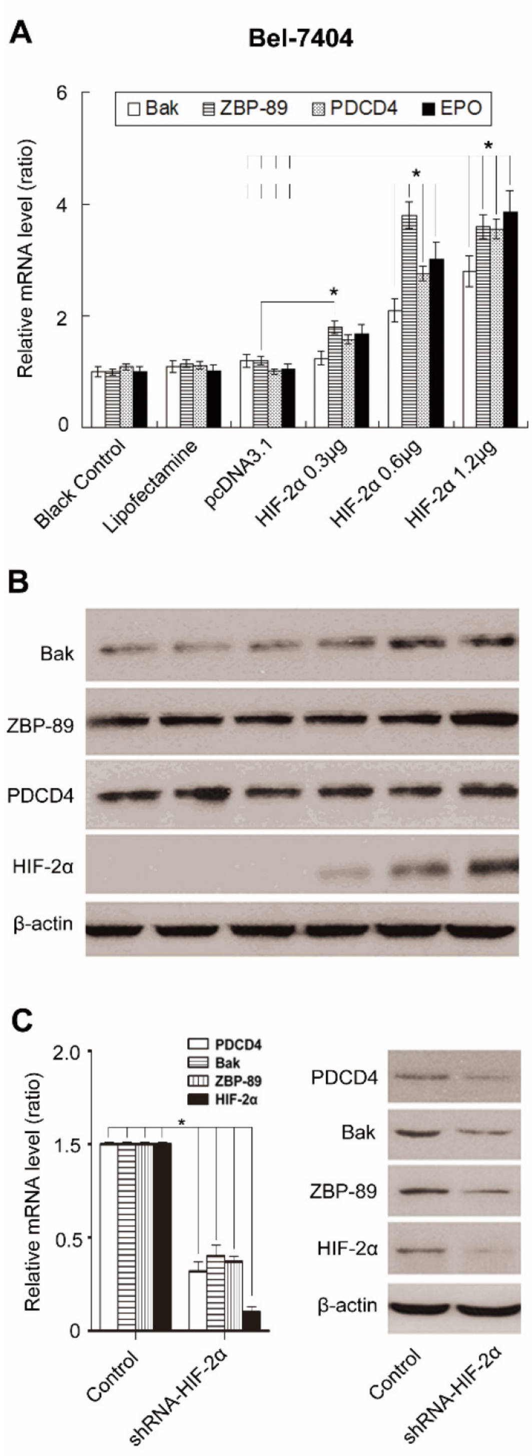

Supplementary Figure S3: Effects of HIF-2α on the expression of Bak, ZBP-89 and PDCD4 in Bel-7404 cells. Real-time PCR **A.** and western blotting **B.** analysis of the expression of Bak, ZBP-89, PDCD4 and erythropoietin (EPO) in Bel-7404 cells (respectively transfected with 0, 0.3, 0.6 and 1.2 μg pcDNA3.1-HIF-2α for 24 h). Real-time PCR and Western blotting analysis of Bak, ZBP-89 and PDCD4 levels in Bel-7404 cells treated with HIF-2α shRNA **C.** β-actin was used as a control. Error bars indicated standard deviation (n=3), ***P*<0.01.

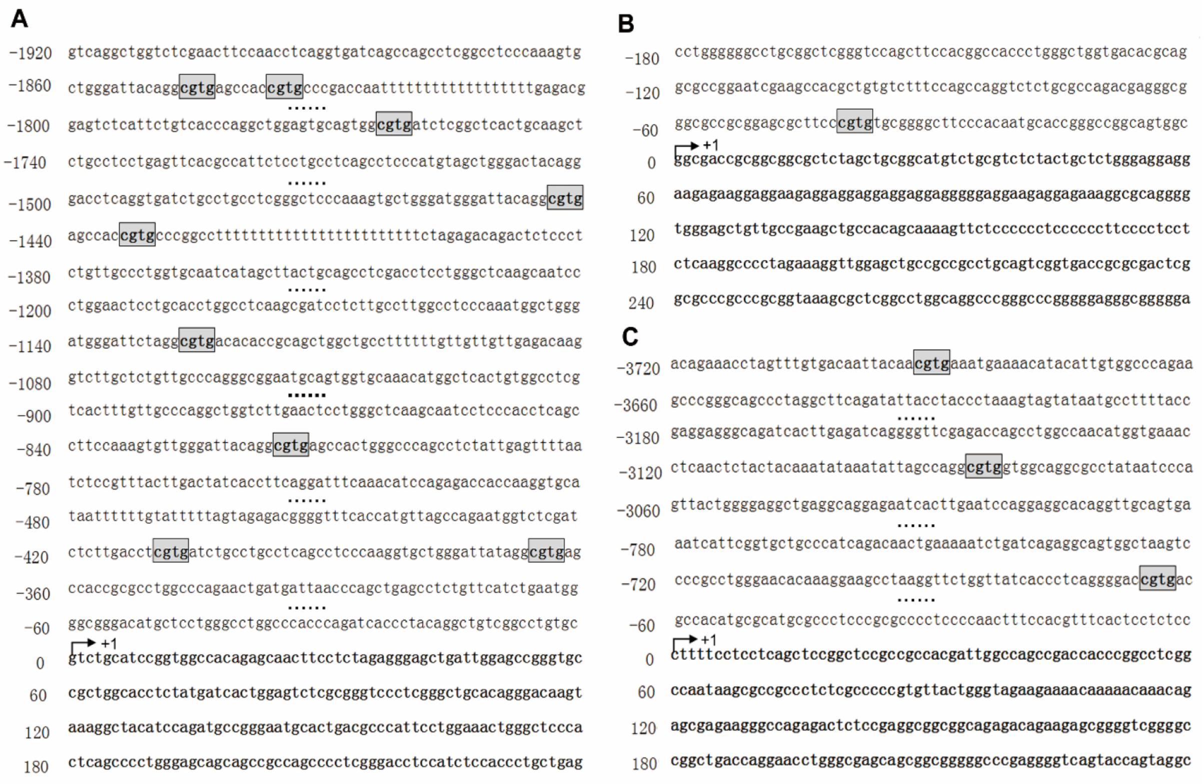

Supplementary Figure S4: Nucleotide sequences of the promoter regions of human Bak (NM_001188.3) A., ZBP-89 (NM_021964) **B.** and PDCD4 (NM_145341.3) **C.** The numbers indicate the base location relative to the start site of the first exon (in bold) that is labeled with an up-arrow. Bold letters represent putative binding sites for HIF-2α and hypoxia response elements (CGTG) that were identified by TRANSFAC (http://www.biobase.international.com).

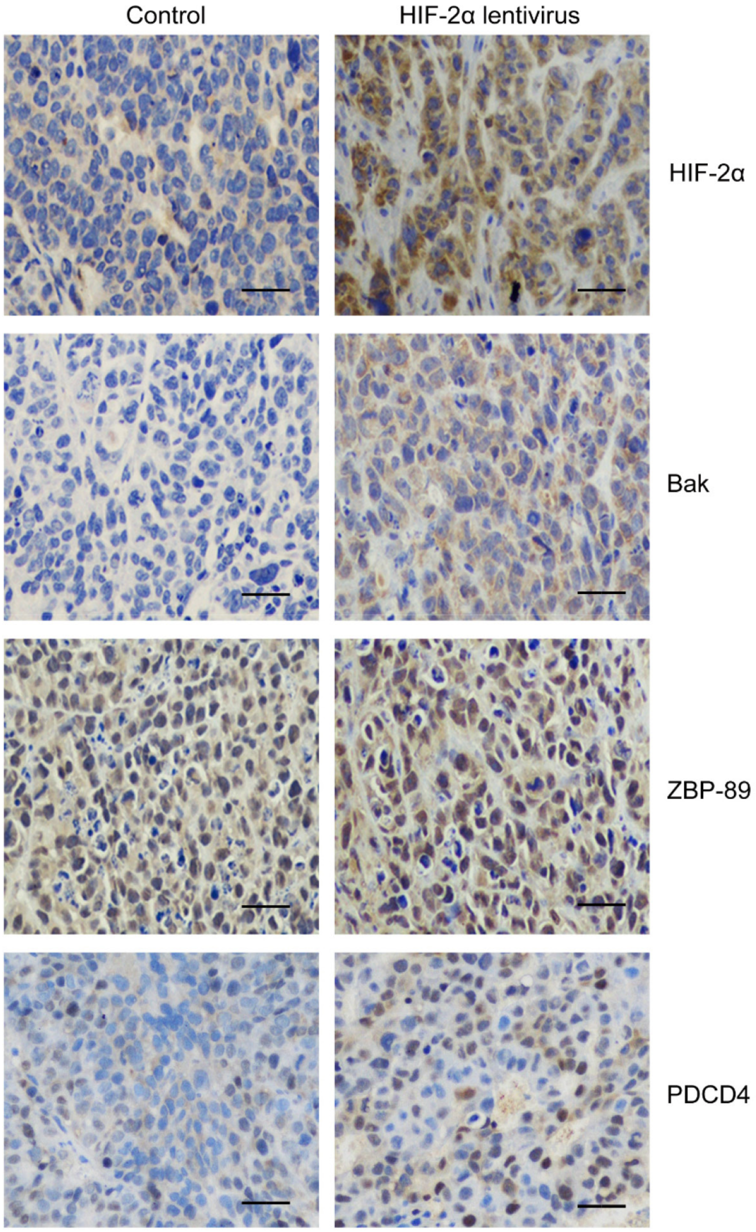

Supplementary Figure S5: Effects of HIF-2α on Bak, ZBP-89 and PDCD4 expression *in vivo*. Tumors were cut into sections and immunohistochemistry staining was performed. The levels HIF-2α, ZBP-89, Bak and PDCD4 proteins were increased in the HIF-2α-lentivirus group, compared with the control (×200). Scale bar, 10mm.
